# Study protocol: double-blind, randomized, prospective, placebo controlled parallel group phase II study to investigate the effect of glycerol phenylbutyrate (GPB) on neurofilament light chain (NfL) levels in patients with corticobasal syndrome (CBS)

**DOI:** 10.1186/s42466-026-00504-5

**Published:** 2026-07-03

**Authors:** Carla Palleis, Endy Weidinger, Sylvia Maaß, Melanie Linke, Matthias Brendel, Sophia Stöcklein, Amani Al Tawil, Ulrich Mansmann, Daniel Teupser, Tobias Borst, Ralph Heimke-Brinck, Bernhard Hemmer, Günter U. Höglinger, Helen Bidner, Mikael Simons, Johannes Levin

**Affiliations:** 1 Department of Neurology, LMU University Hospital, LMU Medizin, Ludwig-Maximilians-Universität München, Munich, Germany; 2 German Center for Neurodegenerative Diseases (DZNE) Munich, Munich, Germany; 3 Munich Cluster for Systems Neurology (SyNergy), Munich, Germany; 4 Department of Neurology, TUM School of Medicine and Health, Technical University Munich, Munich, Germany; 5 Department of Nuclear Medicine, LMU University Hospital, LMU Medizin, Ludwig-Maximilians-Universität München, Munich, Germany; 6 Department of Radiology, LMU University Hospital, LMU Medizin, Ludwig-Maximilians-Universität München, Munich, Germany; 7 Institut Für Medizinische Informationsverarbeitung, Biometrie Und Epidemiologie (IBE), LMU Medizin, Ludwig-Maximilians-Universität München, Munich, Germany; 8 Institute of Laboratory Medicine, LMU University Hospital, LMU Medizin, Ludwig-Maximilians-Universität München, Munich, Germany; 9 Pharmacy of the University Hospital Erlangen, Uniklinikum Erlangen, Erlangen, Germany; 10 TUM School of Medicine and Health, Muenchner Studienzentrum (MSZ), Technical University Munich, Munich, Germany; 11 Institute for Stroke and Dementia Research (ISD), LMU University Hospital, LMU Medizin, Ludwig-Maximilians-Universität München, Munich, Germany; 12 Department of Neurology, LMU University Hospital, LMU Medizin, Ludwig-Maximilians-Universität München, Munich, Germany Marchioninistraße 15, 81377

**Keywords:** Corticobasal syndrome, Neurodegeneration, Neurofilament light chain, Glycerol phenylbutyrate, Phase II clinical trial

## Abstract

**Introduction:**

Corticobasal syndrome (CBS) is a rare progressive neurodegenerative disorder, with no disease-modifying treatments currently available. The most common underlying pathology is a 4-repeat tauopathy. Neurofilament light chain (NfL) is a biomarker of neuronal damage and has shown potential as a measure of disease progression. Glycerol phenylbutyrate (GPB), a prodrug of phenylbutyric acid, has demonstrated potential neuroprotective properties in preclinical studies on tauopathies. This phase II clinical trial will investigate the effects of GPB on NfL levels in CBS patients. The primary objective is to assess the efficacy of GPB in reducing NfL levels over 26 weeks compared to placebo as well as safety and tolerability of GPB. Secondary objectives include evaluating changes in clinical scales.

**Methods and analysis:**

This is an investigator-initiated double-blind, randomized, placebo-controlled, parallel-group phase II clinical trial, performed in two German university hospitals. A total of 32 patients with CBS will be enrolled and randomized to receive either GPB or placebo. The primary outcome is the change in NfL levels between baseline and 26 weeks as well as safety and tolerability of GPB. Secondary outcomes are changes in clinical scores. Exploratory analyses involve pharmacokinetics, changes in the metabolomic, proteomic and lipidomic profiles and imaging outcomes, such as MRI and microglia-PET.

**Ethics and dissemination:**

The study protocol has been approved by the lead ethics committee at LMU Munich and conforms to the ethical principles outlined in the Declaration of Helsinki and Good Clinical Practice (GCP) guidelines.

**Perspectives:**

If successful, this clinical trial could identify a novel therapeutic approach for slowing disease progression in CBS, contributing to a broader understanding of GPB’s therapeutic potential.

**Clinical trial registration:**

The clinical trial has been registered in ClinicalTrials.gov (NCT05983588) and due to Transition in the Clinical Trials Information System (CTIS; EUCT No. 2024-516897-31-00, date of transition: 2024-09-26).

## Background

Corticobasal syndrome (CBS) is a neurodegenerative disorder characterized by a combination of cortical and extrapyramidal symptoms including apraxia, dystonia and cognitive impairment [1, 2]. While CBS describes the clinical manifestation, corticobasal degeneration (CBD) refers to one of the corresponding neuropathological entities. The majority of CBS patients have either a Progressive Supranuclear Palsy (PSP)-pathology, a CBD-pathology or an Alzheimer Disease (AD)-pathology, each approximately 30%, while rare causes include TDP-43 proteinopathy [3–5]. Tau is a microtubule-associated protein with several functions in the neuronal cytoskeleton. In four-repeat (4R-) tauopathies such as CBD or PSP, cytoplasmic inclusions predominantly composed of tau-protein are found. The pathophysiological mechanisms leading to cell death are not deciphered to the absolute detail, but include post-translational modifications, misfolding and aggregation of tau. Therefore, tau protein and specifically pathological tau aggregation is a highly promising and well-accepted target for the development of disease modifying targets [6]. Clinical diagnoses of CBS has a reported prevalence of 4.9 to 7.3 cases per 100,000 individuals in the general population [7]. CBS is a sporadic disease with typical onset in the fifth to seventh decade and average survival time of 6–8 years following symptom onset [4, 5, 8]. 

The effect of treatment options on the symptoms of patients with CBS is very limited. The therapies currently recommended are symptomatic in nature, relying on physiotherapy, speech therapy and pharmacological modulation of dopaminergic systems in the affected brain regions for akinetic‐rigid symptoms [9, 10]. Despite its devastating effects, no disease-modifying treatments exist for CBS. As CBS is rapidly leading to loss of independence and ultimately to reduced survival, effective disease modifying treatment is urgently needed.

In addition to pathological aggregation of tau-protein, neuroinflammation is key to the pathogenesis in neurodegenerative diseases with tau- and/or AD-pathology [11, 12]. Misfolded and aggregated proteins can trigger an innate immune response characterized by the release of inflammatory mediators. Moreover, abnormally activated microglia have shown to be part of the seeding process that generates and amplifies protein aggregates [13].

Research over the last few years has strengthened the link between microglial-driven neuroinflammation, 4-repeat (4R) tau pathology and disease progression in CBS, indicating that in CBS due to 4R tau, microglial activation is spatially coupled to tau pathology and organized along large‑scale functional networks rather than single regions and dynamically related to disease course [14–16]. 

All in all, modulation of neuroinflammatory responses is an accepted and promising therapeutic strategy. Therefore, orally bioavailable brain penetrant agents addressing both neuroinflammation and protein aggregation are highly promising for the treatment of neurodegenerative therapies in general.

Phenylbutyrate is a clinically approved, orally available and blood–brain barrier penetrant drug, which exhibits pleiotropic effects [17]. It is a class 1 histone deacetylase inhibitor and it also upregulates heat shock proteins and acts as a small molecular chaperone. It can prevent misfolded protein aggregation and reduce endoplasmic reticulum stress. Phenylbutyrate has shown efficacy in several animal studies including models of tauopathies. Phenylbutyrate rescued progressive tauopathy and cognitive changes in two different tau transgenic mouse models [18, 19]. The proposed mechanisms by which phenylbutyrate regulates immune functions is through the inhibition of histone deacetylase activity, by promoting the acetylation of lysine residues present in histones. In our own work, we can show that treatment of mice with phenylbutyrate enhances the capacity of microglia to phagocytose and clear myelin debris after demyelinating injury [20]. Further, in a mouse model of P301S tau transgenic mice we could show a significant difference of NfL levels and levels of microglial activation as measured by 18 kDa translocator protein positron-emission-tomography (TSPO-PET) with [^18^F]GE-180 and immunohistochemical analysis after treatment with phenylbutyrate vs. placebo (patent: “Oral Phenylbutyrate for Treatment of Human 4-Repeat Tauopathies” (PCT/EP2024/053388) filed by LMU Munich). Together, there is strong evidence that phenylbutyrate can modulate microglial function by enhancing their phagocytic activity, most likely by epigenetic mechanisms. These mechanisms have already shown promising results in human neuroinflammatory disease [21]. In the CENTAUR trial phenylbutyrate has shown first evidence of efficacy in amyotrophic lateral sclerosis [22]. Of note, the CENTAUR results could not be confirmed in the phase III trial PHOENIX (as reported, in press releases and company statements). 

One promising approach for monitoring disease progression in CBS and other neurodegenerative disorders is the use of biomarkers. Neurofilament light chain (NfL), a structural protein in neurons, is released into the bloodstream upon neuronal damage and its levels have been shown to correlate with disease severity and progression across a range of neurodegenerative conditions [23, 24]. In CBS, elevated levels of NfL have been associated with rapid disease progression, making it a promising biomarker for clinical trials [16, 25].

In the investigator-initiated PROFIL study, presented in this protocol, we plan to test the hypothesis that glycerol phenylbutyrate (GPB) – which is a pro-drug of phenylbutyric acid – modulates immune responses implicated in neurodegenerative processes in CBS, by assessing the efficacy of GPB in reducing plasma NfL levels in patients with CBS over a 26-week treatment period, compared to placebo. In a phase II proof-of-concept study targeting neuroinflammatory mechanisms, an imaging-based marker of inflammation would, in principle, represent an ideal primary endpoint. Accordingly, we considered TSPO-PET, which allows in vivo assessment of microglial activation in CBS [14, 15], as a potential primary outcome measure. However, due to the current lack of robust longitudinal TSPO-PET data, reliable effect size estimates and power calculations are not available. This methodological limitation precluded the use of TSPO-PET as a primary endpoint. Consequently, TSPO-PET has been defined as a prespecified exploratory endpoint in this study.

## Methods and analysis

### Objectives

The primary objective is to assess the efficacy of glycerol phenylbutyrate vs. placebo in reducing the levels of NfL during 26 weeks of exposure to glycerol phenylbutyrate as well as safety and tolerability of glycerol phenylbutyrate in patients with CBS.

The secondary objective is to assess longitudinal changes in clinical scales, including motor and cognitive outcomes, between V1 and V3 comparing placebo- vs. verum-treated patients.

Primary Endpoints for Safety and Tolerability are safety, incidence of adverse reactions, safety laboratory evaluation (basic clinical chemistry), vital signs (blood pressure, heart rate, temperature), physical and neurological examination, survival time and survival rate during the study period, tolerability, and number of subjects (and % of the intention-to-treat population), who discontinue the clinical trial due to adverse reactions.

In absence of established scales for clinical assessment of CBS, clinical scales include Movement Disorder Society (MDS) Unified Parkinson’s Disease Rating Scale (UPDRS) – motor part [26], PSP-rating scale (PSP-RS) [27], PSP-Clinical deficit scale (CDS) [28], CBFS [29], DATE [30], Montreal Cognitive Assessment (MoCA) [31], Schwab and England Activities of Daily Living (SEADL), Clinical global impression severity scale (CGI) [32] and PSP-Quality of Life (PSP-QoL) [33].

Additional exploratory analyses beyond the predefined trial endpoints will be conducted in a subset of patients who agreed to participate in the voluntary exploratory sample collection or procedures, including examining pharmacokinetics, changes in the metabolomic, proteomic and lipidomic profiles and imaging outcomes, such as MRI and TSPO-PET with [^18^F]GE-180 [14, 15] and [^18^F]DPA-714.

### Study design and setting

This is a double-blind, randomized, placebo-controlled, phase II clinical trial with a parallel-group design. The trial will be conducted at two centers in Germany and will include 32 patients diagnosed with CBS. Patients will be randomized in a 1:1 ratio to receive either the investigational medicinal product (IMP) GPB or placebo. The treatment duration is 26 weeks, with follow-up visits scheduled at 13 and 26 and 30 weeks (and optional at 6 and 19 weeks). Patients are enrolled at the participating study sites at the university hospitals of the Ludwig-Maximilians University Munich and the Technical University Munich.

### Participants

#### Inclusion criteria

- Age ≥ 18 years
- Diagnosis of clinical possible or probable CBS based on CBD criteria [1] or PSP-CBS [2]
- No regular use of GPB in the 6 months prior to Visit 1 (V1)
- Capability and willingness to comply with the procedures of the clinical trial
- Women of childbearing potential must be non-lactating, have a negative pregnancy test, and either be surgically sterile or use a highly effective contraceptive method (failure rate < 1% per year), with hormonal contraception supplemented by a barrier method (preferably a male condom), while unreliable methods such as periodic abstinence, withdrawal, spermicides alone, or lactational amenorrhoea are not permitted.
- A stable regimen for at least 1 month prior to V1 and no foreseeable need to change the regimen throughout the 26 week treatment period for
  1. drugs acting against Parkinsonism (e.g. Levodopa, Dopamine-Agonists, Amantadine and MAO-B-Inhibitors)
  2. Other central nervous system (CNS)-active substances including e.g. antidepressants and antidementia drugs

#### Exclusion criteria

- Diagnosis of other neurodegenerative diseases
- Alzheimer’s pathology as confirmed by β-amyloid-(Aβ)-PET or reduced Aβ_1-42_ in cerebrospinal fluid (CSF), with regard to the heterogeneity of the possible underlying neuropathology (see above) and in order to increase the homogeneity of the population of CBS-patients (note: phosphorylated tau 181 and Aβ_1-42_/Aβ_1-40_ ratio are measured in exploratory analyses but not used for eligibility)
- Participation in another clinical trial involving administration of an IMP within 1 month or 5 half-lives of the investigational medicinal product prior to V1
- Known hypersensitivity to glycerol phenylbutyrate or its further components, or to drugs with a similar chemical structure or to any of the components of the placebo
- Treatment with valproic acid, haloperidol, or probenecid
- A physical or psychiatric condition (e.g. frontal lobe syndrome, psychotic disorder or major depression), which at the investigator’s discretion may put the subject at risk, may confound the trial results or may interfere with the subject’s participation in this clinical trial
- Persistent abuse of medication, drugs or alcohol
- Current or planned pregnancy or breast-feeding in females
- Other severe medical conditions upon the discretion of the investigator

### Intervention

Participants will be assigned to one of two treatment arms:

- **GPB Group**: Participants will receive GPB (RAVICTI®, 1.1 g/ml oral liquid), starting with 3 ml/day (1.5 ml split between morning and evening doses) for the first 3 weeks, and increasing to 6 ml/day (3 ml split between morning and evening doses) for the remaining 23 weeks.
- **Placebo Group**: Participants will receive a matching placebo with the same dosing schedule as the GPB group.

#### Compound

The IMP GPB is a triglyceride containing molecules of PBA linked to a glycerol backbone. GPB (RAVICTI® Immedica Pharma AB, Stockholm, Sweden) is a pro-drug of PBA. Upon oral ingestion, PBA is released from the glycerol backbone in the gastrointestinal tract by pancreatic lipases. GPB has a marketing authorization for inborn urea cycle disorders (UCDs). As our study participants do not have UCDs, we orientate ourselves regarding the dosage for the PROFIL trial on the CENTAUR trial where phenylbutyrate showed initially efficacy in the context of a neurodegenerative disease (motoneuron disease) [22]. The dose may be reduced to 3 ml/day or 1 ml/day in case of intolerable side effects. Side effects of phenylbutyrate intake in the CENTAUR trial are reported to be mainly gastrointestinal (diarrhea, nausea, salivary hypersecretion and abdominal discomfort).

Verum (GPB) and matching placebo are produced by the pharmacy of Universitätsklinikum Erlangen, Erlangen, Germany. 

The intervention verum or placebo will be taken twice daily. Participants will be instructed to document each dose in a daily diary.

### Randomization and blinding

Randomization will be conducted using a block randomization method to ensure balance between the treatment groups. Both the participants and the investigators will be blinded to the treatment allocation. Randomization codes will be generated by an independent statistician (TUM School of Medicine and Health, Münchner Studienzentrum) and held by the central pharmacy (University of Erlangen, Erlangen, Germany), which will dispense the blinded medication.

### Study procedures and assessments

Participants will undergo the following assessments at each study visit:

- **Screening (Visit V0)**: Informed consent, demographic data collection, medical history, concomitant medication, physical and neurological examinations, vital signs and laboratory tests (routine clinical chemistry, pregnancy tests for women of childbearing potential).
- **Baseline (Visit V1, Day 0)**: NfL sampling, clinical scales (MDS-UPDRS Part III, PSP-RS, PSP-CDS, CBFS, DATE, MoCA, SEADL, CGI-s, PSP-QoL), physical and neurological examinations, vital signs, blood sampling, pharmakokinetic (PK) blood sampling, CSF sampling, MRI and TSPO
- **Follow-up Visits (Visits V2-V4, Weeks, 13, 26, and 30)**: NfL sampling, repeat assessments of clinical scales and safety monitoring (vital signs, laboratory values and AEs), blood sampling, PK blood sampling, CSF sampling (at week 26), MRI (at week 26) and TSPO (at week 26)
- **Optional Visits (Optional visits O1 and O2, weeks 6 and 19)**: NfL sampling, repeat assessments of clinical scales PK sampling, blood sampling. It is not considered as a violation of the protocol, if patients opt out of voluntary exploratory samples/procedures.

**Fig. 1:**
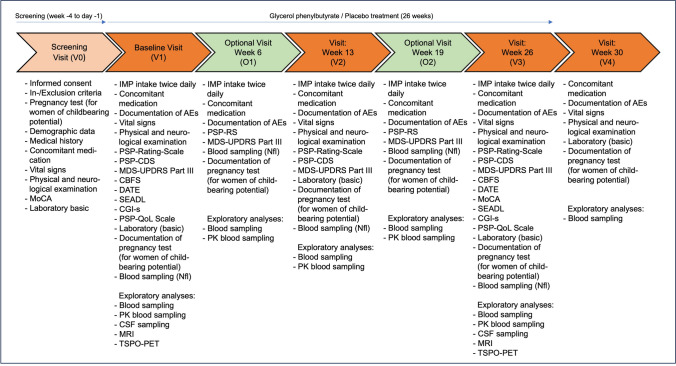
Outline of the PROFIL study from the study protocol: Visit schedule to time scale. AE: Adverse events, CBFS: Cortical Basal Ganglia Functional Scale, CDS: Clinical Deficit Scale, CGI-s: Clinical Global Impression – severity scale, CSF: Cerebrospinal fluid, DATE: Dementia Apraxia Test, IMP: Investigational Medicinal Product, MDS-UPDRS: Movement Disorders Society Unified Parkinson’s Disease Rating Scale, MoCA: Montreal Cognitive Assessment, MRI: Magnetic Resonance Imaging, NfL: Neurofilament Light Chain, PK: Pharmacokinetics, PSP: Progressive Supranuclear Palsy, QoL: Quality of Life, SEADL: Schwab and England Activities of Daily Living, TSPO-PET: Translocator Protein PET imaging

The detailed outline of the study is displayed in Fig. 1.

### Statistical analysis

#### Sample size calculation

The sample size was calculated to detect a 60% reduction in plasma NfL levels between the GPB and placebo groups, with a power of 80% and a significance level of 5%. Based on previous studies, a standard deviation of 0.46 on the log-transformed scale was assumed. Allowing for a dropout rate of 20%, a total of 32 patients (16 per group) will be enrolled.

#### Statistical methods

Longitudinal changes in NfL levels will be analyzed using a linear mixed-effects model, with treatment group, time and their interaction as fixed effects. Random intercepts and slopes will be included to account for individual variability. Missing data will be handled using the assumption that data are missing at random. For sensitivity analyses, per-protocol and intention-to-treat populations will be analyzed. Exploratory analyses will include pharmacokinetic modeling, changes in lipidomic, proteomic and metabolomic profiles and comparisons of MRI and TSPO-PET imaging between the two treatment groups.

Primary Statistical Endpoints:

- The longitudinal changes in the Levels of Neurofilament light chain between V1 and V3 comparing placebo- vs. verum-treated patients
- Tabular description of the multiple safety and tolerability endpoints (physical and neurological examination, safety laboratory evaluation (basic clinical chemistry), adverse events, vital signs, survival rates and survival time) without specific hypothesis testing.

Secondary Statistical Endpoints

- Analyses of the longitudinal change in the secondary read-outs (clinical scales (MDS-UPDRS motor part, PSP-RS, PSP-CDS, CBFS, DATE, MoCA, SEADL, CGI-s, PSP-QoL) between V1 and V3 comparing placebo- vs. verum-treated patients

### Safety monitoring

An independent Data Monitoring Committee (DMC) will monitor the safety of participants and trial progress throughout the clinical trial. AEs will be collected at each study visit and SAEs will be reported immediately to the sponsor. GPB is known to have a favorable safety profile, but the study will closely monitor gastrointestinal side effects (e.g., diarrhea, nausea) and other potential adverse events (e.g., hepatotoxicity, electrolyte imbalances). 

### Trial registration

The clinical trial has been registered in ClinicalTrials.gov (NCT05983588) and due to Transition in the Clinical Trials Information System (CTIS; EUCT No. 2024-516897-31-00, date of transition: 2024-09-26).

## Perspective

NfL is emerging as a promising biomarker for neurodegenerative diseases due to its sensitivity in detecting neuronal damage [16, 24, 34, 35]. The choice of NfL as the primary outcome measure in this trial reflects its potential to capture disease-modifying effects over a relatively short period. By measuring NfL levels longitudinally in CBS patients, we aim to determine whether GPB can slow disease progression, as reflected by reductions in NfL.

The study design, including the use of a placebo control and double blinding, ensures that any observed effects can be attributed to GPB. The inclusion of multiple clinical scales will allow for a comprehensive evaluation of GPB’s effects on both motor and cognitive outcomes. The exploratory analyses of metabolomic, proteomic, and lipidomic profiles, along with advanced imaging modalities (MRI and TSPO-PET) will provide additional insights into the mechanisms underlying GPB’s effects. The exploratory analysis of plasma pharmacokinetics of phenylbutyrate will be of particular interest, as it will generate essential data on systemic exposure and interindividual variability in patients with CBS, thereby supporting exposure–response analyses and informing dose selection, endpoint interpretation, and trial design in subsequent, larger studies.

If successful, this trial will provide the first evidence that GPB can slow disease progression in CBS, a devasting neurodegenerative condition for which no effective treatments currently exist [10]. The findings could pave the way for larger trials and expand the use of GPB to other neurodegenerative disorders characterized by neuroinflammation.

## Conclusion

This investigator-initiated phase II clinical trial will assess the efficacy and safety of GPB in reducing plasma NfL levels and improving clinical outcomes in patients with CBS. Given the urgent need for disease-modifying treatments for neurodegenerative diseases, the results of this trial could have significant implications for the treatment of CBS and related conditions.

## Data Availability

No datasets were generated or analysed during the current study.
